# Seizures as onset symptoms and rapid course in preschool children with subacute sclerosing panencephalitis

**DOI:** 10.1002/brb3.2051

**Published:** 2021-02-05

**Authors:** Shuang Liao, Min Zhong, Nan Zou, Tingsong Li, Li Jiang

**Affiliations:** ^1^ Department of Neurology Children’s Hospital of Chongqing Medical University Ministry of Education Key Laboratory of Child Development and Disorders National Clinical Research Center for Child Health and Disorders (Chongqing) International Science and Technology Cooperation Base of Child Development and Critical Disorders Chongqing Key Laboratory of Pediatrics Chongqing China

**Keywords:** children, clinical manifestation, prognosis, subacute sclerosing panencephalitis

## Abstract

**Background:**

The clinical features and outcomes of subacute sclerosing panencephalitis (SSPE) in younger children are different from those of adults, leading easily to misdiagnosis during the early stage. So far, there are limited data related to SSPE in preschool children.

**Methods:**

In order to summarize the clinical data and evolution of SSPE in preschool children and to expand the phenotypes of SSPE, the medical charts of preschool patients diagnosed with SSPE were retrospectively reviewed and analyzed; the clinical outcomes of the enrolled cases were evaluated and followed up.

**Results:**

Overall, we included three cases in the study. Their onset age was 5 years and 2 months, 4 years and 3 months, and 4 years and 2 months, respectively. All patients presented drop attacks or jerks as the onset symptom, and one patient had concurrent gait disturbance. Atypical periodic complexes on electroencephalography (EEG) were recorded in all patients. The brain magnetic resonance imaging (MRI) findings of two cases showed demyelinating lesions predominantly on the white matter. The neurological conditions of all cases deteriorated rapidly. Two children died at 21 months and 6 months after onset, respectively. The other case progressively developed vegetative status and akinetic mutism within 4 months.

**Conclusions:**

In younger children, the characteristic features of SSPE may be seizures and gait instability as onset manifestations, atypical periodic complexes on EEG, and rapid worsening of neurological conditions.

## Graphical abstract

Onset symptoms of seizures or gait instabilities are common in preschool children with SSPE. The neurological conditions deteriorate rapidly at follow‐up. Atypical periodic complexes on EEG are usual for the younger children with SSPE.

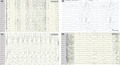

## 1 Introduction

Subacute sclerosing panencephalitis (SSPE) is rare, progressive, and fatal degenerative encephalitis caused by slow measles virus infection in the central nervous system. It usually occurs 7–10 years after measles infection, and the latency varies from 1 month to 27 years (Cruzado et al., 2002). Gutierrez et al., (2010) divided the progressive clinical course of SSPE into four stages, with the initial presentation as personality changes, academic failure in school, and abnormal behaviors. Comparatively, one study (Yilmaz et al., 2006) showed that the onset symptoms in 77.8% (7 out of 9) of cases aged <4 years were myoclonic seizures, rather than cognitive decline. The present data suggest that the clinical features and evolution of SSPE in younger children may be different from that in older age groups.

One survey demonstrated that the incidence of SSPE was 1 in 1,367 for children aged <5 years after they had contracted measles (Wendorf et al., 2017), which was much higher than 6.5–11/100,000, the prevalence of overall populations with measles infection (Bellini et al., 2005). However, there have been limited data related to SSPE in younger children. Therefore, expanding the knowledge on the clinical characteristics and evolution of SSPE in younger children remains imperative. In the present study, we summarize the clinical data of three preschool children diagnosed with SSPE, aiming to accurately recognize SSPE in younger childhood earlier.

## 2 MATERIALS AND METHODS

We retrospectively reviewed the medical charts of all children diagnosed with SSPE and followed their clinical evolution. All diagnoses were established between January 2017 and January 2020 in Children's Hospital of Chongqing Medical University (CHCMU), the largest tertiary children's hospital in Southwest China. The diagnosis fulfilled Dyken's criteria: clinical history and elevated cerebrospinal fluid (CSF) antibody titers as the two major markers, and typical electroencephalography (EEG) as the minor marker (Gutierrez et al., 2010). Antimeasles immunoglobulin G (IgG) in both CSF and serum was tested using enzyme‐linked immunosorbent assay (ELISA).

The clinical data and auxiliary tests included demographics, initial symptoms, neurological manifestations and clinical course, EEG, cranial magnetic resonance imaging (MRI), responsiveness to antiepileptic drugs (AEDs), history of measles vaccination, and infection. This study was approved by the CHCMU ethics committee. Consent was obtained from all the patients’ legal guardians.

## 3 RESULTS

In total, three individuals were diagnosed with SSPE, and all of them were preschool children. The clinical characteristics and evolution are summarized in Table 1. The clinical course was evaluated based on Gutierrez et al. (Gutierrez et al., 2010)

**Table 1:** Summary of the clinical features of the three cases

	Case 1	Case 2	Case 3
Onset age/sex	5 yr 2 mo/male	4 yr 3 mo/female	4 yr 2 mo/male
Initial symptoms	Drop attack, gait disturbance	Drop attack, jerk	Drop attack
Neurological manifestations			
Seizures	myoclonus, absence	Myoclonus, tonic seizures, atonic seizures	Atonic seizures
Cognitive decline	+	+	+
Ataxia	+	+	+
Tremor	+	+	+
Pyramidal signs	+	+	−
Involuntary movement	+	−	+
Age at measles vaccination	8 mo	−	8 mo
Age at measles infection	−	2 yr 1 mo	6 mo
Antimeasles IgG (CSF/serum)/age	1:1 600(CSF)/1:100 000(Serum)/5 yr 5 mo OB(+) (CSF)	1:1 600(CSF)/1:1,000 000(Serum)/4 yr 7 mo	1:1 600(CSF)/1:100 000(Serum)/4 yr 4 mo
(Time after onset) EEG	25 d: NB, PC with asynchronous and asymmetric triphasic waves predominantly on the frontal area. 1 mo 20 d: SB; PC consisting of SW or continuous sharp waves lasting for 5 s maximally, followed by occasional suppression.	3 mo: SB; PC with asynchronous, asymmetric, and continuous sharp waves with duration of 0.5–8 s, followed by suppression.	10 d: NB; PC with slow and sharp waves every 10–150 s; 1.5–2 Hz sharp and SW predominantly in the frontal area in the interval of PC. 22 d: SB; PC with SW lasting for 0.8–1.2 s; electrical status epilepticus during sleep
(Time after onset) MRI	1 mo: Normal; 3 mo 10 d: Bilateral and diffuse hyperintensity in the cerebral white matter and corpus callosum on T2 imaging.	2 mo: Normal; 3 mo 17 d: Patchy lesions with iso T1 and long T2 signal in subcortical area in the bilateral frontal lobe and periventricular white matter.	20 d: Normal 3 mo: Normal.
AEDs/outcomes	LEV + CZP/weekly seizures	VPA + LEV+CZP/no response	VPA + CZP+LTG + KD/no response
Last follow‐up (time after onset)	Vegetative status and akinetic mutism (4 mo).	Died from respiratory complication and dystonia status (21 mo).	Died from respiratory complication (6 mo).

Abbreviations: CZP, clonazepam; d, days; KD, ketogenic diet; LEV, levetiracetam; LTG, lamotrigine; mo, month; NB, normal background; OB, oligoclonal band; PC, periodic complexes; SB, slow background; SW, slow waves; VPA, valproic acid; yr, years.

### 3.1 Clinical history

#### 3.1.1 Case 1

A 5‐year, 3‐month‐old boy was referred to CHCMU for gait instability, repeating drop attacks, and jerks lasting for a month. The initial symptoms were unstable sitting and a tendency to tumble easily while walking, then jerks of the right limbs manifested, which progressively developed to absence and myoclonus of the four limbs five to >30 times daily. Other forms of seizures were not observed. His cognition and motor systems were normal before the symptoms onset and deteriorated within 2 months after onset, which was characterized as no active speech and no response to external stimuli. His motor system symptoms were aggravated from unstable standing initially to being unable to sit without assistance. Limb tremor, ataxia, involuntary movement, hypertonia, and extrapyramidal signs (Stage 2D) were pronounced at 2 months after the onset.

**Figure 1:**
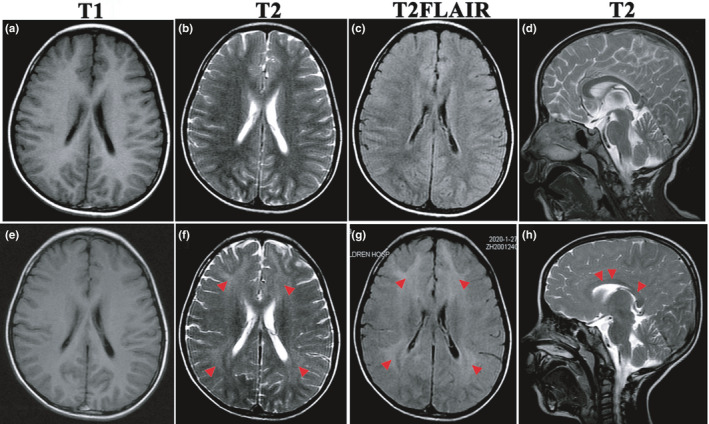
Dynamic presentation of brain MRI of Case 1. The MRI was normal at 1 month after the onset (a–d) and demonstrated bilateral and diffuse lesions in the cerebral white matter and corpus callosum (f–h, red arrowheads) 2 months later (e–h)

His previous growth and development history were unremarkable. His older sister was in good health. He had received his measles vaccination when he was 8 months old on plan, and had never been exposed to measles infection. After admission, CSF infection etiology testing, which included bacterial culture and ELISA for herpes simplex virus, coxsackievirus, enterovirus (EV)‐71, and Epstein–Barr virus IgG antibodies, was negative. The blood/urine screening tests (amino acids in the blood and urine, organic acids in the urine, lactate in the blood) for metabolic diseases were also negative. Due to the presence of acute encephalopathy and involuntary movements, autoimmune encephalitis (AE) was initially suspected. However, AE‐associated anti‐IgGs in the serum and CSF, which had been tested twice, were negative. Meanwhile, the oligoclonal band was positive in the CSF. When the initial EEG recording was retrospectively reviewed, we found atypical periodic complexes (PC). Subsequently, the typical PC became more obvious over the disease course. The brain MRI was normal initially and showed demyelinating lesion at 2 months after onset (Figure 1). At 3 months after onset, serum and CSF revealed increased antimeasles antibody, confirming the diagnosis of SSPE. Levetiracetam (LEV) and clonazepam (CZP) were introduced, and the myoclonic jerks are partially under control, with persistent weekly seizures. However, his neurodevelopment progressively deteriorated to vegetative status and akinetic mutism (Stage 4) at the last follow‐up (4 months after onset).

#### 3.1.2 Case 2

A 4‐year, 5‐month‐old girl, previously well and developmentally normal, was admitted to hospital with complaint of paroxysmal drop attacks for 2 months, followed by repetitive jerks for >20 days. The clinical course became aggravated following onset. Ataxia, slurred speech, limb weakness, and bilateral tonic seizures or tonic–clonic seizures were also observed at more than 20 days after onset (Stage 2B). Valproic acid (VPA) was given initially, but was not effective. She developed drowsiness, inability to sit without support, and pyramidal signs with normal muscular tension within 3 months. Transient tonic seizures and jerks frequently occurred 10 to >30 times daily. VPA, LEV, and CZP were entirely ineffective. Tremor, hypertonia, coma, and frequent seizures (Stage 3) appeared at 3.5 months of the course. CZP was stopped due to pneumonia during hospitalization, and intriguingly, the seizures were reduced to >10 times a day after the pneumonia had been treated.

The patient had no siblings. Measles vaccination had not been received on schedule, and she had measles infection when she was 2 years and 1 month old. As with Case 1, CSF infection etiology testing and blood/urine screening tests were both negative. Auditory evoked potential and fundus examination were normal. Brain MRI on day 2 (3 months after onset) in hospital revealed high‐intensity signals in the white matter of the bilateral frontal and periventricular white matter on T2 imaging. Therefore, we considered it central nervous system demyelinating disease, and sequential therapy with methylprednisolone and prednisone was introduced, but the effects were not obvious. Combining the progressively declined neurological condition and periodic complexes on EEG, we tested antimeasles IgG at 4 months after onset, and then, SSPE was established. The patient died from respiratory complication and dystonia status when she was 6 years and 1 month old.

#### 3.1.3 Case 3

A 4‐year, 2‐month‐old boy was admitted to hospital for frequent drop attacks for a week. Disturbance of consciousness was not observed in the first week. There were no abnormal neurological signs at admission. EEG showed generalized epileptic discharges, which led to clinical suspicion of Doose syndrome. Tremor appeared over 2 weeks, and disturbance of consciousness, irritability, and gibberish occurred over 1 month (Stage 2B). VPA, nitrazepam (NZP), and lamotrigine (LTG) were introduced sequentially, which led to about 50% reduction in seizures, with >20 times daily persisting. Subsequently, he was put on the ketogenic diet, which was entirely ineffective. He developed drowsiness, hypotonia, and frequent seizures at about 2 months after onset (Stage 2C).

His developmental milestones were normal. His older sister did not have an unusual medical history. The patient had measles infection when he was 6 months old, and nevertheless received the measles vaccination when he was 8 months old. EEG at onset showed atypical PC. Brain MRI was performed twice within 2 months, and both were normal. As in Case 1, CSF infection etiology testing, CSF/serum AE antibodies, and blood/urine screening tests were all negative. He was positive for antimeasles antibody IgG at 2 months, which confirmed the diagnosis of SSPE. He died from respiratory complication when he was 4 years and 8 months old.

### 3.2 EEG

**Figure 2:**
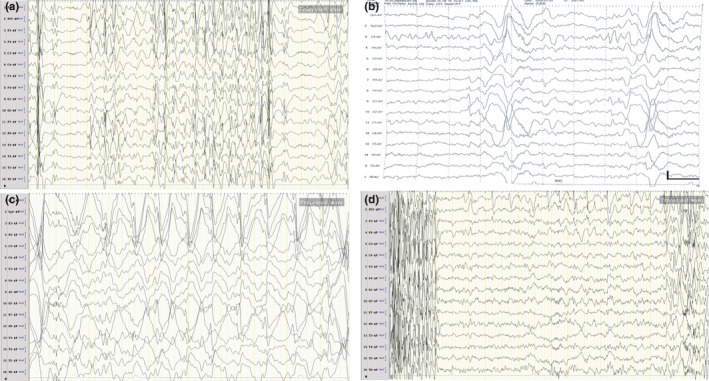
EEG recordings. (a) EEG recording of Case 2 at 3 months after onset showing complexes consisting of asynchronous, asymmetric, and continuous sharp waves with duration of 2–7 s, followed by suppression for 1–2 s. Displayed at 20 s per page and sensitivity of 10 μV. (b) EEG recording of Case 1 at 25 days after onset showing atypical periodic complexes composed of asynchronous and asymmetric triphasic waves predominantly in the frontal area (scale bar: 100 μV/s). (c) Electrical status epilepticus during sleep in Case 3 at 22 days after onset. (d) EEG recording of Case 3 at 10 days after the beginning demonstrating prolonged 1.5–2 Hz sharp and slow waves predominantly in the frontal area during the interval of the periodic complexes. Displayed at 20 s per page and sensitivity of 10 μV

In total, we reviewed five EEG records from the three patients. In spite of the presence of the typical periodic complexes (Gutierrez et al., 2010) in all patients, at least one atypical feature was recorded for each case: Asymmetric and asynchronous complexes in Case 1 and 2; complexes consisting of continuous sharp waves in Cases 1 and 2 (Figure 2a), as well as sharp and slow waves in Case 3; the duration of complexes was >3 s in all cases; prolonged complexes followed by suppression for 1–4 s in Cases 1 and 2 (Figure 2a); and periodic, regional, and single triphasic waves in Case 1 (Figure 2b). In addition, Case 3 had electrical status epilepticus during sleep (Figure 2c), and prolonged 1.5–2 Hz sharp and slow waves predominantly in the frontal area in the interval of the complexes (Figure 2d). Nonepileptic jerks were recorded in Case 2.

## 4 DISCUSSION

Compared with the clinical features of common cases aged 8–11 years (Gutierrez et al., 2010), the present study showed that SSPE in preschool children has distinct clinical characteristics and evolution: The onset symptoms were seizures presenting as jerks or drop attacks, and neurological condition deterioration was more rapid. Additionally, all cases showed minimal responsiveness to AEDs.

The initial symptoms of all three cases were drop attacks with or without gait instability, rather than cognitive decline, which is in line with the reported toddler cases (Kasinathan et al., 2019; Kamate et al., 2012) and preschool patients (Magurano et al., 2017). Similarly, Yilmaz et al., (2006) reported that seizures were the onset manifestation in 88.9% of cases (8 out of 9) aged <4 years. Besides the above symptoms, confusion, somnolence, and unsteady gait (Holt et al., 2016) (in a 3‐year‐old), as well as gait instability (in a 5‐year‐old boy) (Saini et al., 2016) have also been reported. Two of our patients died 21 months and 6 months after onset, respectively. The remaining child progressively developed rigidity and was barely conscious within 5 months. Likewise, the majority of the reported toddler cases died within 12 months after onset (Yilmaz et al., 2006; Kasinathan et al., 2019; Kamate et al., 2012). It appears that, in younger children, cases aged >5 years have a lifespan shorter than 3.8 years (45 days–12 years) (Guler et al., 2015). All the data suggest that both the onset symptoms and clinical course in younger children are different from those of adults or older children.

In the present case series, only Case 1 showed no history of measles virus infection and had received routine vaccination by schedule. Similarly, SSPE has been reported in two other children without measles infection (Kamate and Detroja, 2018). Based on epidemiological evidence that the measles vaccine virus does not cause SSPE (Campbell et al., 2007), the patients are believed to have subclinical or undiagnosed measles infection. Therefore, the absence of measles virus infection should not exclude the diagnosis of SSPE.

The periodic Radermecker complexes on EEG, as a diagnostic criterion, were recorded in all three cases. However, none of the cases were suspected as SSPE based on the EEG recordings initially. This may have been due to the atypical EEG findings in all of the cases. Moreover, the onset presentations, such as jerks, ataxic and tonic seizures, as well as encephalopathy, may lead to the diagnosis of some epilepsy syndromes, for example, progressive myoclonic epilepsy, Lennox‐Gastaut syndrome (Demir et al., 2013), and Doose syndrome (Magurano et al., 2017). Compared with previously reported atypical EEG (Ekmekci et al., 2005), electrical status epilepticus was recorded in the present study. Our results highlight that atypical period complexes on EEG may be more common in younger children, and SSPE should be kept in mind, particularly for the cases with fulminant encephalopathy.

All three cases showed normal cranial MRI at onset, and Cases 1 and 2 had subsequent development of T2 hyperintensity on the white matter. Case 3 never demonstrated any abnormal signal on MRI until 2 months after onset. Demyelinating lesions on brain MRI have also been reported in 5‐month‐old and 8‐year‐old cases (Dey and Ghosh, 2020). Although brain neuroimaging is specific to neither the diagnostic criteria nor the clinical severity (Mekki et al., 2019), our results assert that SSPE should be suspected in the acquired demyelinating diseases with periodic complexes on EEG, even in the absence of history of measles infection.

To date, there is no curative treatment for SSPE. Most treatments are symptomatic interventions. In our series, all patients received two AEDs minimally; the ketogenic diet was even introduced for Case 3. However, they were not responsive to the treatment. In addition, we recorded nonepileptic jerks in our case series, which highlights the importance of differentiating the seizure type using ictal EEG to avoid the potential adverse effects caused by unnecessary AEDs.

Collectively, preschool children with SSPE have characteristic onset symptoms presenting as seizures or gait instabilities and more rapid progressive deterioration. Atypical periodic Radermecker complexes on EEG may be more common than in other age groups and be an early diagnostic clue for SSPE in younger children. EEG recordings, MRI findings, and clinical courses are not always parallel during the clinical evolution. Based on the lack of curative treatment for SSPE caused by measles infection, increasing vaccination coverage in countries with high incidence of measles epidemics remains the most effective means of eradicating this devastating disease.

## CONFLICTS OF INTERESTS

The authors declare that they have no competing interests.

## AUTHOR CONTRIBUTIONS

Shuang Liao collected the clinical data and drafted the manuscript. Min Zhong established the diagnosis and performed the follow‐up. Nan Zou wrote the reports of the cases and followed up the patients. Tingsong Li made conception and design of the work and revised the manuscript. Li Jiang interpreted the data and revised the manuscript.

## Data Availability

All the anonymized data generated or used in the study could be reached by request from any qualified investigator.
